# A new pseudopolymorph of berberine chloride: crystal structure and Hirshfeld surface analysis

**DOI:** 10.1107/S2056989022003309

**Published:** 2022-04-05

**Authors:** Tatiana Kornilova, Viktor Glebov, Raúl Castañeda, Tatiana V. Timofeeva

**Affiliations:** a New Mexico Highlands University, 1005 Diamond Ave., Las Vegas NM, 87701, USA

**Keywords:** crystal structure, stacking inter­actions, methanol solvate, Hirshfeld surface analysis

## Abstract

A new pseudopolymorph of berberine, 9,10-dimeth­oxy-5,6-di­hydro-2*H*-7λ^5^-[1,3]dioxolo[4,5-*g*]iso­quinolino­[3,2-*a*]isoquinolin-7-ylium chloride methanol monosolvate, C_20_H_18_NO_4_^+^·Cl^−^·CH_3_OH, was obtained during co-crystallization of berberine chloride with malonic acid from methanol. The berberine cations form dimers, which are further packed in stacks. The title structure was compared with other reported solvates of berberine chloride: its dihydrate, tetra­hydrate, and ethanol solvate hemihydrate. Hirshfeld analysis was performed to show the inter­molecular inter­actions in the crystal structure of the title compound, and its fingerprint plots were compared with those of the already studied solvates.

## Abstract 2

The crystal structure of methanol solvate of berberine chloride, 9,10-dimeth­oxy-5,6-di­hydro-2*H*-7λ^5^-[1,3]dioxolo[4,5-*g*]iso­quinolino­[3,2-*a*]isoquinolin-7-ylium chloride methanol monosolvate, was determined and its Hirshfeld surface analysis was performed.

## 1. Chemical context

The ability of co-crystals and polymorphs of active pharmaceutical ingredients (APIs) to change their physicochemical properties without modification of their biological activity has been pointed out in multiple publications, for instance, Shan & Zaworotko (2008 ▸), Bernstein (2002 ▸, 2005 ▸) and Brittain (2009 ▸). Currently, examples presented in the literature demonstrate that some attempts to grow co-crystals of organic compounds, including APIs, with particular coformers (additives) result in the formation of new polymorphs (Song & Cölfen, 2011 ▸). In some cases, the combining of particular compounds with additives can increase the nucleation rate and thus lead to the development of a new crystalline form of the substance. Most likely, the additive suppressed formation of the general form, as a result of which a new polymorph begins to grow (Lee, 2014 ▸). For example, it was reported that combining different additives (trimesic acid, benzoic acid, phthalic acid, isophthalic acid, *etc*) with hexol (Co_4_H_42_N_12_O_18_S_3_), allowed two different polymorphic and one new pseudopolymorphic forms of this substance to be obtained (Mehta *et al.*, 2007 ▸).

New polymorph modifications are often obtained sporadically. For example, the second form of maleic acid was found only recently, in 2006, while the first form was reported in 1881. Inter­estingly, this new form was obtained during co-crystallization of maleic acid with caffeine (Day *et al.*, 2006 ▸). Another example of this phenomenon was demonstrated by the well-known explosive 1,3,5-tri­nitro­benzene, which was co-crystallized with tris­indane. Instead obtaining of a new co-crystal, two new polymorphs of the main compound were discovered (Thallapally *et al.*, 2004 ▸). These examples demonstrate that sometimes applying additives to the compound of inter­est may lead to a new polymorph. In some cases, the polymorph modifications demonstrate improved properties compared to the previously known form of the substance. Kobayashi *et al.* (2000 ▸) compared the dissolution rate and bioavailability of carbamazepine dihydrate and its polymorphs. It was noted that one of the polymorphs showed a higher dissolution rate than the other species.

Berberine, a natural product belonging to the class of alkaloids, is extracted from the leaves, barks, or roots of various plants such as *Coptis chinensis, Hydrastis canadensis, etc*. (Babu *et al.*, 2012 ▸). It was reported that berberine and its derivatives can be highly effective against inflammatory processes (Yeşilada & Küpeli, 2002 ▸), fungi (Silva *et al.*, 2016 ▸), used as anti­oxidants (El-Wahab *et al.*, 2013 ▸), or mutagens (Čerňáková *et al.*, 2002 ▸). Currently, berberine is available as a supplement. Berberine chloride (BCl) is a stable salt of berberine that is soluble in water (Battu *et al.*, 2010 ▸). The primary goal of this study was to obtain co-crystals of berberine chloride with three different acids, glutaric, malonic, and succinic, in an attempt to increase its solubility. In addition, it was inter­esting to follow studies of BCl hydrates (Singh *et al.*, 2018 ▸), demonstrating the mechanical responses of BCl single crystals on cooling and heating. 
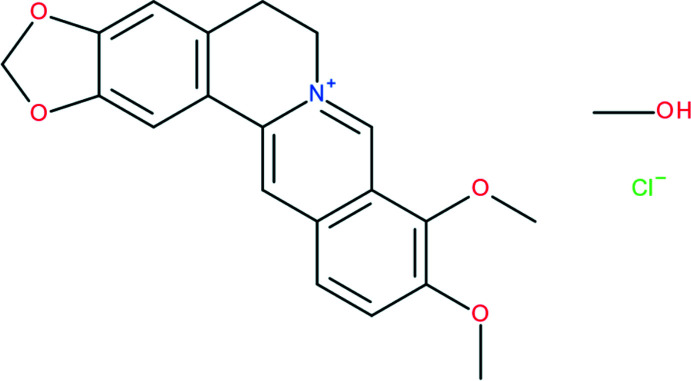


## 2. Crystallization

Berberine chloride (Alfa Aesar, lot No. R25HO28) was co-crystallized with glutaric (Alfa Aesar, lot No. D22Z032), malonic (Alfa Aesar, lot No. 10178800), and succinic (Spectrum, lot No. 1BK0179) acids. A slow evaporation technique was used for all three experiments. A molar ratio 1:1 for each pair was used; the compounds were dissolved separately in 5 mL of methanol with heating and ultrasonication. After that, those solutions were combined together and filtered. Then the three resulting solutions were left for evaporation at room temperature. After 7 days, small yellow needles were collected from the solutions with glutaric and malonic acids. The sample with succinic acid was not suitable for further characterization. The structure characterization showed that samples of BCl with glutaric and malonic acids gave two different species: one with two water mol­ecules and another with one mol­ecule of methanol. The obtained pseudopolymorph with two water mol­ecules had been studied before (Kariuki & Jones, 1995 ▸). Crystals of the new BCl solvate with methanol were very fragile and dissipated very quickly in the air, most probably because of solvent loss. These crystals were used for diffraction studies with necessary precautions.

## 3. Structural commentary

**Figure 1:**
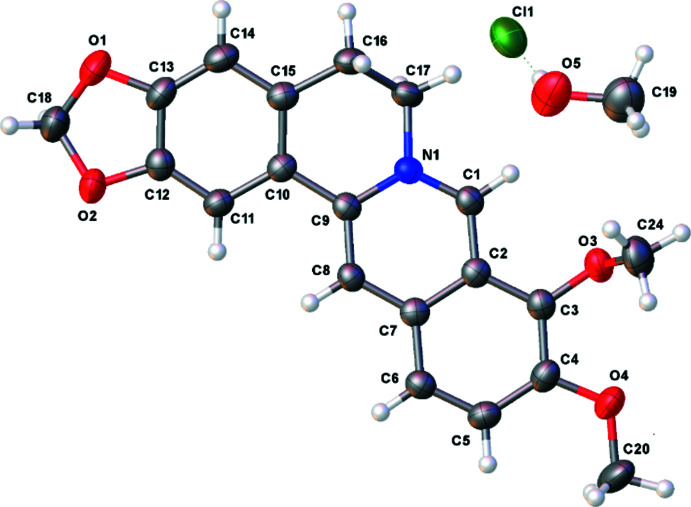
Mol­ecular structure of the title compound with the atom labeling. Displacement ellipsoids are drawn at the 50% level.

Berberine chloride is a quaternary ammonium salt from the group of iso­quinoline alkaloids. The berberine core (Fig. 1 ▸) contains two almost planar aromatic fragments (N1/C1–C9 and C10–C15) with a dihedral angle of 13.91 (4)° between them, which is similar to the corresponding values in other berberine cations presented in Table 1 ▸. The bond lengths and bond angles in the cation are in line with those of previously reported analogues (Kariuki & Jones, 1995 ▸; Singh *et al.*, 2018 ▸). The positions of the single and double bonds (see scheme) correspond to the bond lengths found in our experimental diffraction study. One of the two methyl­ene groups attached to the cation lies almost in the plane of the aromatic ring while the other is nearly perpendicular to it (Fig. 1 ▸). The torsion angles involving these groups are 5.8 (2)° for C20—O4—C4—C5 and −79.29 (18)° for C24—O3—C3—C4.

## 4. Supra­molecular features

**Figure 2:**
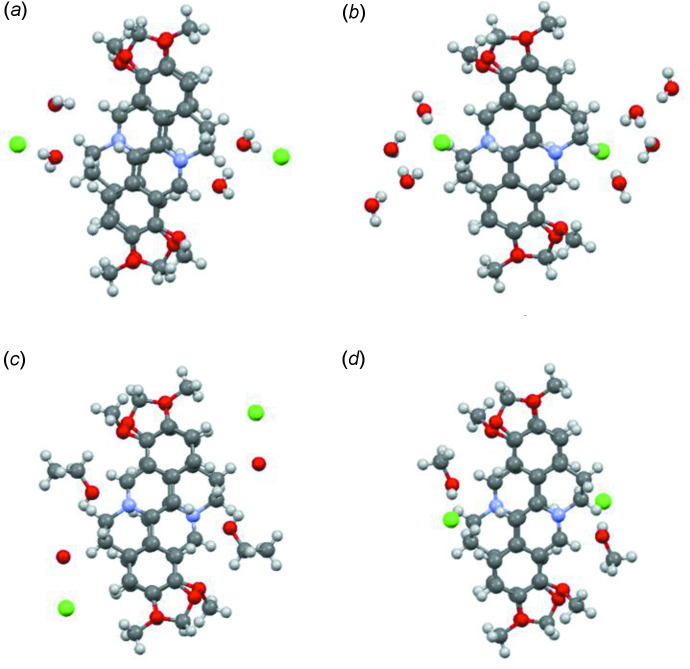
The dimers of berberine cations in the pseudopolymorphs with (*a*) two water mol­ecules, (*b*) four water mol­ecules, (*c*) one mol­ecule of ethanol and 0.5 mol­ecules of water, and (*d*) one methanol mol­ecule (see text for references).

The berberine cations in the structure of the title compound are not involved in the formation of any hydrogen bonds. The only short contact that might be considered as a specific inter­action is the contact of Cl^−^ with the methanol hydrogen atom H5*A* [2.23 (2) Å]. This distance is quite close to the value of 2.079 Å that was presented in the review by Kovács & Varga (2006 ▸). Details of the hydrogen-bond geometry are given in Table 2 ▸. In the crystal, the berberine cations form stacks along the *a-*axis direction. The neighboring cations within the stack are related by inversion (Fig. 2 ▸). The inter­planar distance (only core atoms were included in plane calculation) to the cation related by the symmetry operation −*x* + 1, −*y* + 1, −*z* + 1 is shorter than that to the other cation related by −*x* + 2, −*y* + 1, −*z* + 1, being 3.564 (2) and 3.498 (2) Å, respectively. In general, the crystal packing can be described as ‘stacks that are built of dimers’.

## 5. Database survey

A search of the Cambridge Structural Database (CSD version 5.42, last update November 2020; Groom *et al.*, 2016 ▸) demonstrated the significant inter­est in berberine salts. The structure of BCl dihydrate has been determined three times [XUNFES (Tong *et al.*, 2010 ▸); XUNFES01 (Singh *et al.*, 2018 ▸); XUNFES02 (Fronczek, 2019 ▸)] with almost equal precision. The structure of BCl tetra­hydrate has been determined twice [YUJHAM (Kariuki & Jones, 1995 ▸); YUJHAM01 (Singh *et al.*, 2018 ▸)]. To the best of our knowledge, the only non-solvated berberine salt to be characterized is the iodine deriv­ative (YUJHUG; Kariuki & Jones, 1995 ▸). In addition, BCl ethanol solvate (YUJHIU; Kariuki & Jones, 1995 ▸), as well as berberine iodide monohydrate (KUZSAA; Grundt *et al.*, 2010 ▸), bromide dihydrate (YUJHOA; Kariuki & Jones, 1995 ▸), and sulfate hepta­hydrate (YUJJAO; Kariuki & Jones, 1995 ▸) should be mentioned. The very inter­esting type of behavior exhibited by the BCl dihydrate and tetra­hydrate at different temperatures was described by Singh *et al.* (2018 ▸). Depending on the chosen conditions, the crystals demonstrated unexpected mechanical responses: bending, cracking, and jumping. The explanation for these thermo-mechanical properties was linked to the presence of destabilizing inter­actions between the water mol­ecules.

To estimate the similarities and differences between the crystal structures of pseudopolymorphs of BCl, we compared the hydrogen bonding and mol­ecular packing for the four solvates presented in Table 1 ▸. All of the berberine cations in these structures are arranged in stacks, the space group for all compounds except for the dihydrate is *P* 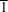 ; for the dihydrate, the space group is *C*2/*c*. The stacks are formed of the very similar dimers shown in Fig. 2 ▸. Table 1 ▸ demonstrates that the cations in stacks are situated in such a way that the distances between the mean planes (only core atoms were included in plane calculations) of the cations vary by *ca* 0.2 Å. The distances between the centroids of the aromatic rings characterizing the mol­ecular slippage show more diversity than the inter­planar distances.

As in the title structure, the water mol­ecules in the dihydrate and in the ethanol solvate do not form hydrogen bonds with the berberine cation, but make short contacts with the Cl^−^ anion. However, in the crystal structure of the tetra­hydrate, one of the water mol­ecules forms a bifurcated hydrogen bond with the berberine cation.

## 6. Hirshfeld surface analysis

**Figure 3:**
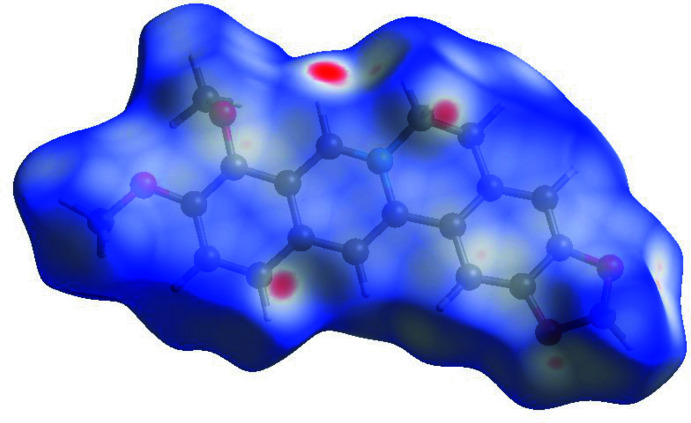
Hirshfeld surface for the berberine cation in the title structure plotted over *d*_norm_ in the range −0.1877 to 1.1413 a.u.

**Figure 4:**
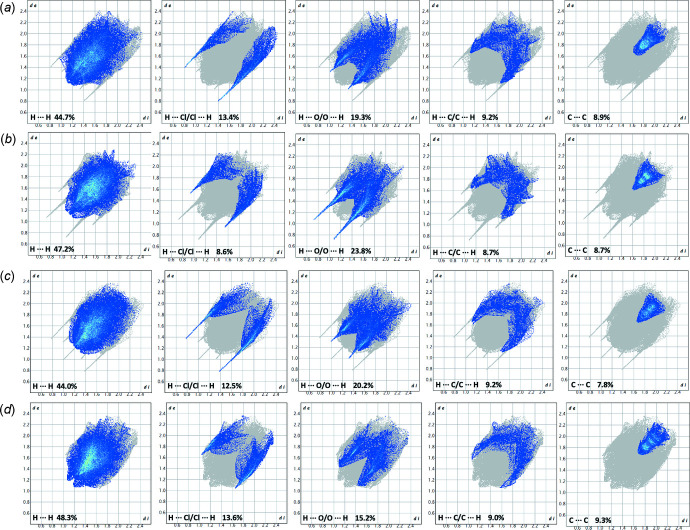
The two-dimensional fingerprint plots for pseudopolymorphs of BCl with (*a*) two water mol­ecules, (*b*) four water mol­ecules, (*c*) one mol­ecule of ethanol and 0.5 mol­ecules of water, and (*d*) one methanol mol­ecule.

The Hirshfeld surface analysis was performed using *Crystal Explorer* (Wolff *et al.*, 2012 ▸). According to the Hirshfeld surface presented in Fig. 3 ▸, the shortest inter­molecular contacts are found for the hydrogen atoms attached to the nitro­gen-containing C1–N1–C17 fragment. Fig. 4 ▸ gives the fingerprint plots for all the pseudopolymorphs presented in Table 1 ▸. There are 15 different types of inter­actions in these crystals between five elements – H, C, N, Cl, and O – from which 60 fingerprint plots can be generated. 20 plots for which the inter­actions contribute above 2% to the Hirshfeld surface are presented in Fig. 4 ▸. In spite of the different number and nature of the solvate/hydrate mol­ecules in the pseudopolymorphs presented, the fingerprint plots allow generalization of the impact of the inter­molecular inter­actions in these structures. In all structures, the H⋯H contacts provide the largest contributions (44.0–48.3%). The presence of H⋯O/O⋯H inter­actions, corresponding to inter­actions between the solvate mol­ecules, is also important (15.2–23.8%). The next highest contribution is by inter­actions involving the Cl^−^ anion (8.6–13.6%). The fingerprint plot for the methanol solvate is different from the others since there are no water mol­ecules in this structure, and no hydrogen bonds between the solvent and berberine cation.

## 7. Refinement

Crystal data, data collection and structure refinement details are summarized in Table 3 ▸. The O-bound H atom was refined freely. All other H atoms were positioned geometrically (C—H = 0.95, 0.98 and 0.98 Å for *sp* ^2^-hybridized, methyl and methyl­ene C atoms, respectively) and refined using a riding model, with *U*_iso_(H) = 1.5*U*_eq_(C) and 1.2*U*_eq_(C) for methyl and other H atoms, respectively.

## Supplementary Material

Crystal structure: contains datablock(s) I. DOI: 10.1107/S2056989022003309/yk2164sup1.cif

Structure factors: contains datablock(s) I. DOI: 10.1107/S2056989022003309/yk2164Isup3.hkl

Supporting information file. DOI: 10.1107/S2056989022003309/yk2164Isup3.cml

CCDC reference: 2161706

Additional supporting information:  crystallographic information; 3D view; checkCIF report

## Figures and Tables

**Table 1:** Selected crystallographic data for berberine chloride pseudopolymorphs

	(C_20_H_18_NO_4_)^+^·Cl^−^·2H_2_O	(C_20_H_18_NO_4_)^+^·Cl^−^·4H_2_O	(C_20_H_18_NO_4_)^+^·Cl^−^·EtOH·0.5H_2_O	(C_20_H_18_NO_4_)^+^·Cl^−^·MeOH
CSD Refcode	XUNFES01	YUJHAM01	YUJHIU	
Space group	*C*2/*c*	*P* 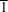	*P* 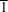	*P* 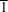
*a* (Å)	27.449 (7)	6.8909 (4)	7.371 (1)	7.332 (2)
*b* (Å)	7.0744 (17)	11.4787 (6)	11.2724 (10)	9.886 (3)
*c* (Å)	21.677 (6)	13.1419 (7)	13.3998 (10)	13.270 (4)
α (°)	90	76.205 (4)	77.587 (7)	93.359 (8)
β (°)	117.695 (7)	89.221 (4)	73.299 (7)	102.703 (8)
γ (°)	90	85.231 (4)	78.228 (8)	92.410 (8)
*Z*	8	2	2	2
ρ (g cm^−3^)	1.454	1.465	1.377	1.434
Dihedral angle between aromatic fragments (°)	13.64 (4)	11.3 (1)	11.0 (1)	13.91 (4)
Mean-plane deviation (Å)	0.185	0.161	0.161	0.196
Distances between mol­ecular planes (Å)	3.5408 (12), 3.6475 (12)	3.4280 (6), 3.5330 (7)	3.4222 (19), 3.4144 (17)	3.5640 (19), 3.4982 (16)
Distances between centroids (Å)	4.2997 (11), 5.1407 (12)	4.3583 (5), 5.1838 (5)	4.6729 (15), 4.5413 (15)	5.9017 (16), 4.3704 (14)

**Table 2:** Hydrogen-bond geometry (Å, °)

*D*—H⋯*A*	*D*—H	H⋯*A*	*D*⋯*A*	*D*—H⋯*A*
O5—H5*A*⋯Cl1	0.84 (2)	2.23 (2)	3.0613 (18)	176 (2)

**Table 3:** Experimental details

Crystal data	
Chemical formula	C_20_H_18_NO_4_^+^·Cl^−^·CH_4_O
*M*_r_	403.84
Crystal system, space group	Triclinic, *P* 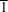
Temperature (K)	100
*a*, *b*, *c* (Å)	7.332 (2), 9.886 (3), 13.270 (4)
α, β, γ (°)	93.359 (8), 102.703 (8), 92.410 (8)
*V* (Å^3^)	935.3 (4)
*Z*	2
Radiation type	Mo *K*α
μ (mm^−1^)	0.24
Crystal size (mm)	0.55 × 0.10 × 0.08

Data collection	
Diffractometer	Bruker APEXII CCD
Absorption correction	Multi-scan (*SADABS*; Bruker, 2016 ▸)
*T*_min_, *T*_max_	0.642, 0.745
No. of measured, independent and observed [*I* > 2σ(*I*)] reflections	14444, 3376, 2867
*R*_int_	0.040
(sin θ/λ)_max_ (Å^−1^)	0.602

Refinement	
*R*[*F* ^2^ > 2σ(*F* ^2^)], *wR*(*F* ^2^), *S*	0.034, 0.095, 1.03
No. of reflections	3376
No. of parameters	260
H-atom treatment	H atoms treated by a mixture of independent and constrained refinement
Δρ_max_, Δρ_min_ (e Å^−3^)	0.25, −0.20

Computer programs: *APEX2* (Bruker, 2016 ▸), *SAINT* (Bruker, 2016 ▸), *SHELXT2014/5* (Sheldrick, 2015*a* ▸), *SHELXL2018/3* (Sheldrick, 2015*b* ▸), *OLEX2* (Dolomanov *et al.*, 2009 ▸).

## supplementary crystallographic information

### Crystal data

**Table:**

C_20_H_18_NO_4_^+^·Cl^−^·CH_4_O	*Z* = 2
*M**_r_* = 403.84	*F*(000) = 424
Triclinic, *P*1	*D*_x_ = 1.434 Mg m^−^^3^
*a* = 7.332 (2) Å	Mo *K*α radiation, λ = 0.71073 Å
*b* = 9.886 (3) Å	Cell parameters from 6433 reflections
*c* = 13.270 (4) Å	θ = 2.5–25.3°
α = 93.359 (8)°	µ = 0.24 mm^−^^1^
β = 102.703 (8)°	*T* = 100 K
γ = 92.410 (8)°	Needle, yellow
*V* = 935.3 (4) Å^3^	0.55 × 0.10 × 0.08 mm

### Data collection

**Table:**

Bruker APEXII CCD diffractometer	2867 reflections with *I* > 2σ(*I*)
φ and ω scans	*R*_int_ = 0.040
Absorption correction: multi-scan (SADABS; Bruker, 2016)	θ_max_ = 25.3°, θ_min_ = 1.6°
*T*_min_ = 0.642, *T*_max_ = 0.745	*h* = −8→8
14444 measured reflections	*k* = −11→11
3376 independent reflections	*l* = −15→15

### Refinement

**Table:**

Refinement on *F*^2^	0 restraints
Least-squares matrix: full	Hydrogen site location: mixed
*R*[*F*^2^ > 2σ(*F*^2^)] = 0.034	H atoms treated by a mixture of independent and constrained refinement
*wR*(*F*^2^) = 0.095	*w* = 1/[σ^2^(*F*_o_^2^) + (0.0478*P*)^2^ + 0.2603*P*] where *P* = (*F*_o_^2^ + 2*F*_c_^2^)/3
*S* = 1.03	(Δ/σ)_max_ < 0.001
3376 reflections	Δρ_max_ = 0.25 e Å^−^^3^
260 parameters	Δρ_min_ = −0.20 e Å^−^^3^

### Special details

**Table:**

Geometry. All esds (except the esd in the dihedral angle between two l.s. planes) are estimated using the full covariance matrix. The cell esds are taken into account individually in the estimation of esds in distances, angles and torsion angles; correlations between esds in cell parameters are only used when they are defined by crystal symmetry. An approximate (isotropic) treatment of cell esds is used for estimating esds involving l.s. planes.

### Fractional atomic coordinates and isotropic or equivalent isotropic displacement parameters (Å^2^)

**Table:**

	*x*	*y*	*z*	*U*_iso_*/*U*_eq_	
Cl1	−0.04980 (6)	0.79926 (5)	0.29804 (3)	0.04056 (15)	
O3	0.59675 (15)	0.44604 (11)	0.16673 (8)	0.0296 (3)	
O2	0.84006 (16)	0.65879 (12)	0.92758 (8)	0.0337 (3)	
O4	0.66750 (17)	0.18213 (12)	0.14990 (9)	0.0368 (3)	
O1	0.81836 (18)	0.89074 (12)	0.92490 (9)	0.0389 (3)	
N1	0.67296 (16)	0.65364 (12)	0.45335 (9)	0.0214 (3)	
O5	0.3207 (2)	0.74509 (16)	0.23676 (11)	0.0543 (4)	
H5A	0.217 (3)	0.758 (2)	0.2511 (18)	0.059 (7)*	
C2	0.69666 (19)	0.45102 (15)	0.35149 (11)	0.0219 (3)	
C7	0.76561 (19)	0.38502 (15)	0.44335 (11)	0.0212 (3)	
C10	0.76096 (19)	0.67708 (15)	0.64377 (11)	0.0219 (3)	
C9	0.74277 (19)	0.59387 (15)	0.54597 (11)	0.0209 (3)	
C1	0.6537 (2)	0.58739 (15)	0.36132 (11)	0.0225 (3)	
H1	0.609737	0.633529	0.300595	0.027*	
C3	0.6685 (2)	0.38017 (16)	0.25342 (12)	0.0240 (3)	
C15	0.7548 (2)	0.81869 (15)	0.64324 (12)	0.0248 (3)	
C8	0.78951 (19)	0.46096 (15)	0.53918 (11)	0.0217 (3)	
H8	0.839546	0.418854	0.601054	0.026*	
C4	0.7038 (2)	0.24385 (16)	0.24690 (12)	0.0268 (3)	
C13	0.7967 (2)	0.83456 (16)	0.82525 (12)	0.0286 (4)	
C6	0.8054 (2)	0.24662 (15)	0.43410 (12)	0.0240 (3)	
H6	0.853927	0.200822	0.494398	0.029*	
C12	0.8071 (2)	0.69476 (16)	0.82650 (12)	0.0262 (3)	
C11	0.7888 (2)	0.61277 (15)	0.73828 (12)	0.0246 (3)	
H11	0.794358	0.517079	0.740006	0.030*	
C16	0.7342 (2)	0.88310 (15)	0.54205 (12)	0.0267 (3)	
H16A	0.859043	0.897425	0.526098	0.032*	
H16B	0.680678	0.972788	0.547572	0.032*	
C5	0.7741 (2)	0.17823 (16)	0.33812 (12)	0.0267 (3)	
H5	0.800303	0.085098	0.332994	0.032*	
C14	0.7722 (2)	0.89967 (16)	0.73571 (13)	0.0303 (4)	
H14	0.767092	0.995548	0.735977	0.036*	
C17	0.6085 (2)	0.79463 (15)	0.45553 (12)	0.0256 (3)	
H17A	0.478548	0.792738	0.465500	0.031*	
H17B	0.609059	0.833345	0.388459	0.031*	
C18	0.7981 (3)	0.77680 (18)	0.98451 (13)	0.0389 (4)	
H18A	0.885395	0.789163	1.053243	0.047*	
H18B	0.668620	0.767261	0.994812	0.047*	
C24	0.7346 (3)	0.4840 (2)	0.10979 (13)	0.0397 (4)	
H24A	0.672386	0.519761	0.044460	0.060*	
H24B	0.801969	0.404203	0.095175	0.060*	
H24C	0.823412	0.553809	0.150948	0.060*	
C20	0.6856 (3)	0.03844 (18)	0.13960 (15)	0.0465 (5)	
H20A	0.656566	0.007410	0.066030	0.070*	
H20B	0.598574	−0.007843	0.174415	0.070*	
H20C	0.814203	0.017533	0.171297	0.070*	
C19	0.2949 (3)	0.7269 (2)	0.12882 (16)	0.0553 (5)	
H19A	0.254055	0.811053	0.097614	0.083*	
H19B	0.199487	0.653443	0.102187	0.083*	
H19C	0.413266	0.703462	0.111243	0.083*	

### Atomic displacement parameters (Å^2^)

**Table:**

	*U*^11^	*U*^22^	*U*^33^	*U*^12^	*U*^13^	*U*^23^
Cl1	0.0405 (3)	0.0448 (3)	0.0379 (3)	−0.00321 (19)	0.01065 (19)	0.01218 (19)
O3	0.0315 (6)	0.0354 (6)	0.0231 (6)	0.0089 (5)	0.0065 (5)	0.0056 (5)
O2	0.0441 (7)	0.0364 (6)	0.0203 (6)	0.0081 (5)	0.0064 (5)	−0.0011 (5)
O4	0.0496 (7)	0.0327 (6)	0.0251 (6)	0.0103 (5)	0.0024 (5)	−0.0069 (5)
O1	0.0542 (8)	0.0335 (7)	0.0275 (6)	0.0011 (6)	0.0093 (6)	−0.0088 (5)
N1	0.0221 (6)	0.0208 (6)	0.0229 (7)	0.0019 (5)	0.0077 (5)	0.0035 (5)
O5	0.0457 (8)	0.0675 (10)	0.0444 (8)	0.0259 (7)	−0.0025 (7)	−0.0087 (7)
C2	0.0179 (7)	0.0248 (8)	0.0244 (8)	0.0002 (6)	0.0076 (6)	0.0018 (6)
C7	0.0167 (7)	0.0230 (7)	0.0249 (8)	−0.0006 (6)	0.0071 (6)	0.0020 (6)
C10	0.0187 (7)	0.0227 (8)	0.0247 (8)	−0.0011 (6)	0.0067 (6)	0.0000 (6)
C9	0.0183 (7)	0.0225 (7)	0.0232 (8)	−0.0002 (6)	0.0074 (6)	0.0039 (6)
C1	0.0209 (7)	0.0258 (8)	0.0226 (8)	0.0016 (6)	0.0081 (6)	0.0043 (6)
C3	0.0207 (7)	0.0289 (8)	0.0229 (8)	0.0030 (6)	0.0057 (6)	0.0032 (6)
C15	0.0232 (8)	0.0230 (8)	0.0288 (8)	0.0003 (6)	0.0080 (6)	0.0011 (6)
C8	0.0211 (7)	0.0235 (8)	0.0214 (7)	0.0009 (6)	0.0063 (6)	0.0042 (6)
C4	0.0254 (8)	0.0302 (8)	0.0244 (8)	0.0030 (6)	0.0055 (6)	−0.0032 (7)
C13	0.0286 (8)	0.0299 (8)	0.0263 (8)	−0.0002 (7)	0.0067 (7)	−0.0067 (7)
C6	0.0224 (7)	0.0242 (8)	0.0264 (8)	0.0024 (6)	0.0066 (6)	0.0042 (6)
C12	0.0238 (8)	0.0311 (8)	0.0239 (8)	0.0023 (6)	0.0056 (6)	0.0020 (6)
C11	0.0260 (8)	0.0222 (8)	0.0262 (8)	0.0017 (6)	0.0074 (6)	−0.0003 (6)
C16	0.0300 (8)	0.0198 (7)	0.0324 (9)	0.0022 (6)	0.0109 (7)	0.0034 (6)
C5	0.0260 (8)	0.0231 (8)	0.0312 (8)	0.0029 (6)	0.0072 (7)	−0.0006 (6)
C14	0.0334 (9)	0.0226 (8)	0.0350 (9)	−0.0001 (7)	0.0098 (7)	−0.0034 (7)
C17	0.0288 (8)	0.0215 (8)	0.0285 (8)	0.0066 (6)	0.0091 (7)	0.0054 (6)
C18	0.0527 (11)	0.0392 (10)	0.0248 (9)	0.0106 (8)	0.0086 (8)	−0.0020 (7)
C24	0.0444 (10)	0.0493 (11)	0.0287 (9)	0.0044 (8)	0.0136 (8)	0.0092 (8)
C20	0.0604 (12)	0.0343 (10)	0.0382 (10)	0.0137 (9)	−0.0009 (9)	−0.0133 (8)
C19	0.0539 (13)	0.0633 (14)	0.0491 (12)	0.0147 (11)	0.0112 (10)	0.0001 (10)

### Geometric parameters (Å, º)

**Table:**

O3—C3	1.3692 (18)	C8—H8	0.9500
O3—C24	1.439 (2)	C4—C5	1.413 (2)
O2—C12	1.3800 (19)	C13—C12	1.388 (2)
O2—C18	1.434 (2)	C13—C14	1.365 (2)
O4—C4	1.3577 (19)	C6—H6	0.9500
O4—C20	1.434 (2)	C6—C5	1.375 (2)
O1—C13	1.3771 (19)	C12—C11	1.362 (2)
O1—C18	1.435 (2)	C11—H11	0.9500
N1—C9	1.3985 (19)	C16—H16A	0.9900
N1—C1	1.3277 (19)	C16—H16B	0.9900
N1—C17	1.4913 (18)	C16—C17	1.511 (2)
O5—H5A	0.84 (2)	C5—H5	0.9500
O5—C19	1.403 (2)	C14—H14	0.9500
C2—C7	1.420 (2)	C17—H17A	0.9900
C2—C1	1.402 (2)	C17—H17B	0.9900
C2—C3	1.411 (2)	C18—H18A	0.9900
C7—C8	1.411 (2)	C18—H18B	0.9900
C7—C6	1.415 (2)	C24—H24A	0.9800
C10—C9	1.473 (2)	C24—H24B	0.9800
C10—C15	1.403 (2)	C24—H24C	0.9800
C10—C11	1.418 (2)	C20—H20A	0.9800
C9—C8	1.375 (2)	C20—H20B	0.9800
C1—H1	0.9500	C20—H20C	0.9800
C3—C4	1.384 (2)	C19—H19A	0.9800
C15—C16	1.500 (2)	C19—H19B	0.9800
C15—C14	1.404 (2)	C19—H19C	0.9800
			
C3—O3—C24	113.45 (12)	C12—C11—C10	116.81 (14)
C12—O2—C18	104.11 (12)	C12—C11—H11	121.6
C4—O4—C20	117.96 (13)	C15—C16—H16A	109.5
C13—O1—C18	104.21 (12)	C15—C16—H16B	109.5
C9—N1—C17	120.12 (12)	C15—C16—C17	110.85 (12)
C1—N1—C9	122.41 (12)	H16A—C16—H16B	108.1
C1—N1—C17	117.41 (12)	C17—C16—H16A	109.5
C19—O5—H5A	108.6 (16)	C17—C16—H16B	109.5
C1—C2—C7	118.11 (13)	C4—C5—H5	119.4
C1—C2—C3	121.13 (14)	C6—C5—C4	121.25 (14)
C3—C2—C7	120.74 (13)	C6—C5—H5	119.4
C8—C7—C2	118.12 (13)	C15—C14—H14	121.5
C8—C7—C6	123.47 (14)	C13—C14—C15	117.05 (15)
C6—C7—C2	118.41 (13)	C13—C14—H14	121.5
C15—C10—C9	120.13 (13)	N1—C17—C16	110.76 (12)
C15—C10—C11	120.57 (13)	N1—C17—H17A	109.5
C11—C10—C9	119.28 (13)	N1—C17—H17B	109.5
N1—C9—C10	117.99 (13)	C16—C17—H17A	109.5
C8—C9—N1	117.48 (13)	C16—C17—H17B	109.5
C8—C9—C10	124.53 (13)	H17A—C17—H17B	108.1
N1—C1—C2	121.67 (14)	O2—C18—O1	107.08 (13)
N1—C1—H1	119.2	O2—C18—H18A	110.3
C2—C1—H1	119.2	O2—C18—H18B	110.3
O3—C3—C2	119.09 (13)	O1—C18—H18A	110.3
O3—C3—C4	121.25 (13)	O1—C18—H18B	110.3
C4—C3—C2	119.55 (14)	H18A—C18—H18B	108.6
C10—C15—C16	118.82 (13)	O3—C24—H24A	109.5
C10—C15—C14	120.94 (14)	O3—C24—H24B	109.5
C14—C15—C16	120.22 (14)	O3—C24—H24C	109.5
C7—C8—H8	118.9	H24A—C24—H24B	109.5
C9—C8—C7	122.17 (14)	H24A—C24—H24C	109.5
C9—C8—H8	118.9	H24B—C24—H24C	109.5
O4—C4—C3	115.86 (14)	O4—C20—H20A	109.5
O4—C4—C5	124.33 (14)	O4—C20—H20B	109.5
C3—C4—C5	119.81 (14)	O4—C20—H20C	109.5
O1—C13—C12	109.63 (14)	H20A—C20—H20B	109.5
C14—C13—O1	128.09 (15)	H20A—C20—H20C	109.5
C14—C13—C12	122.27 (14)	H20B—C20—H20C	109.5
C7—C6—H6	119.9	O5—C19—H19A	109.5
C5—C6—C7	120.19 (14)	O5—C19—H19B	109.5
C5—C6—H6	119.9	O5—C19—H19C	109.5
O2—C12—C13	109.29 (13)	H19A—C19—H19B	109.5
C11—C12—O2	128.37 (14)	H19A—C19—H19C	109.5
C11—C12—C13	122.33 (14)	H19B—C19—H19C	109.5
C10—C11—H11	121.6		
			
O3—C3—C4—O4	1.7 (2)	C3—C2—C7—C6	0.0 (2)
O3—C3—C4—C5	−178.63 (13)	C3—C2—C1—N1	177.44 (13)
O2—C12—C11—C10	−178.08 (14)	C3—C4—C5—C6	1.2 (2)
O4—C4—C5—C6	−179.13 (14)	C15—C10—C9—N1	−15.96 (19)
O1—C13—C12—O2	−1.39 (18)	C15—C10—C9—C8	164.13 (14)
O1—C13—C12—C11	179.46 (13)	C15—C10—C11—C12	0.5 (2)
O1—C13—C14—C15	179.53 (15)	C15—C16—C17—N1	−52.38 (16)
N1—C9—C8—C7	−1.2 (2)	C8—C7—C6—C5	178.34 (13)
C2—C7—C8—C9	2.1 (2)	C13—O1—C18—O2	22.69 (17)
C2—C7—C6—C5	−1.2 (2)	C13—C12—C11—C10	0.9 (2)
C2—C3—C4—O4	177.95 (13)	C6—C7—C8—C9	−177.41 (13)
C2—C3—C4—C5	−2.4 (2)	C12—O2—C18—O1	−23.50 (17)
C7—C2—C1—N1	−1.2 (2)	C12—C13—C14—C15	0.9 (2)
C7—C2—C3—O3	178.12 (12)	C11—C10—C9—N1	165.41 (12)
C7—C2—C3—C4	1.8 (2)	C11—C10—C9—C8	−14.5 (2)
C7—C6—C5—C4	0.6 (2)	C11—C10—C15—C16	177.13 (13)
C10—C9—C8—C7	178.67 (13)	C11—C10—C15—C14	−1.3 (2)
C10—C15—C16—C17	35.95 (18)	C16—C15—C14—C13	−177.85 (14)
C10—C15—C14—C13	0.5 (2)	C14—C15—C16—C17	−145.62 (14)
C9—N1—C1—C2	2.2 (2)	C14—C13—C12—O2	177.46 (14)
C9—N1—C17—C16	38.12 (17)	C14—C13—C12—C11	−1.7 (2)
C9—C10—C15—C16	−1.5 (2)	C17—N1—C9—C10	−3.74 (18)
C9—C10—C15—C14	−179.90 (13)	C17—N1—C9—C8	176.18 (12)
C9—C10—C11—C12	179.17 (13)	C17—N1—C1—C2	−175.01 (13)
C1—N1—C9—C10	179.13 (12)	C18—O2—C12—C13	15.39 (17)
C1—N1—C9—C8	−0.9 (2)	C18—O2—C12—C11	−165.53 (16)
C1—N1—C17—C16	−144.61 (13)	C18—O1—C13—C12	−13.23 (17)
C1—C2—C7—C8	−0.89 (19)	C18—O1—C13—C14	168.00 (17)
C1—C2—C7—C6	178.67 (13)	C24—O3—C3—C2	104.41 (16)
C1—C2—C3—O3	−0.5 (2)	C24—O3—C3—C4	−79.29 (18)
C1—C2—C3—C4	−176.85 (13)	C20—O4—C4—C3	−174.50 (15)
C3—C2—C7—C8	−179.54 (13)	C20—O4—C4—C5	5.8 (2)

### Hydrogen-bond geometry (Å, º)

**Table:**

*D*—H···*A*	*D*—H	H···*A*	*D*···*A*	*D*—H···*A*
O5—H5*A*···Cl1	0.84 (2)	2.23 (2)	3.0613 (18)	176 (2)

## References

[bb1] Babu, H. N. R., Thriveni, H. N. & Vasudeva, R. (2012). *J. Nat. Prod. Plant Resour.* **2**, 540–544.

[bb2] Battu, S. K., Repka, M. A., Maddineni, S., Chittiboyina, A. G., Avery, M. A. & Majumdar, S. (2010). *AAPS J.* **11**, 1466–1475. 10.1208/s12249-010-9520-y PMC2974104 20842541

[bb3] Bernstein, J. (2002). *Polymorphism in Molecular Crystals*. New York: Oxford University Press.

[bb4] Bernstein, J. (2005). *Cryst. Growth Des.* **5**, 1661–1662.

[bb5] Brittain, H. G. (2009). *Polymorphism in Pharmaceutical Solids.* New York: Informa, Healthcare.

[bb6] Bruker (2016). *APEX2*, *SAINT* and *SADABS*. Bruker AXS Inc., Madison, Wisconsin, USA.

[bb7] Čerňáková, M., Košt’álová, D., Kettmann, V., Plodová, M., Tóth, J. & Dřímal, J. (2002). *BMC Complement. Altern. Med.* **2**: 2. 10.1186/1472-6882-2-2 PMC101396 11943071

[bb8] Day, G. M., Trask, A. V., Motherwell, W. S. & Jones, W. (2006). *Chem. Commun.* pp. 54–56. 10.1039/b513442k 16353090

[bb9] Dolomanov, O. V., Bourhis, L. J., Gildea, R. J., Howard, J. A. K. & Puschmann, H. (2009). *J. Appl. Cryst.* **42**, 339–341.

[bb10] El-Wahab, A. E. A., Ghareeb, D. A., Sarhan, E. E., Abu-Serie, M. M. & El Demellawy, M. A. (2013). *BMC Complement. Altern. Med.* **13**: 218. 10.1186/1472-6882-13-218 PMC4016550 24007270

[bb11] Fronczek, F. (2019). Personal communication (refcode XUNFES02). CCDC, Cambridge, England.

[bb12] Groom, C. R., Bruno, I. J., Lightfoot, M. P. & Ward, S. C. (2016). *Acta Cryst.* B**72**, 171–179. 10.1107/S2052520616003954 PMC4822653 27048719

[bb13] Grundt, P., Pernat, J., Krivogorsky, B., Halverson, M. A. & Berry, S. M. (2010). *Acta Cryst.* E**66**, o2585–o2586. 10.1107/S1600536810036664 PMC2983369 21587567

[bb14] Kariuki, B. M. & Jones, W. (1995). *Acta Cryst.* C**51**, 1234–1240.

[bb15] Kobayashi, Y., Ito, S., Itai, S. & Yamamoto, K. (2000). *Int. J. Pharm.* **193**, 137–146. 10.1016/s0378-5173(99)00315-4 10606776

[bb16] Kovács, A. & Varga, Z. (2006). *Coord. Chem. Rev.* **250**, 710–727.

[bb17] Lee, E. H. (2014). *Asian J. Pharm. Sci.* **9**, 163–175.

[bb18] Mehta, G., Sen, S. & Venkatesan, K. (2007). *CrystEngComm*, **9**, 144–151.

[bb19] Shan, N. & Zaworotko, M. J. (2008). *Drug Discovery*, **13**, 440–446. 10.1016/j.drudis.2008.03.004 18468562

[bb20] Sheldrick, G. M. (2015*a*). *Acta Cryst.* A**71**, 3–8.

[bb21] Sheldrick, G. M. (2015*b*). *Acta Cryst.* C**71**, 3–8.

[bb22] Silva, A. R. da, de Andrade Neto, J. B., da Silva, C. R., Campos, R. de S., Rde, S., Costa Silva, R. A., Freitas, D. D., do Nascimento, F. B., de Andrade, L. N., Sampaio, L. S., Grangeiro, T. B., Magalhães, H. I., Cavalcanti, B. C., de Moraes, M. O. & Nobre Júnior, H. V. (2016). *Antimicrob. Agents Chemother.* **60**, 3551–3557. 10.1128/AAC.01846-15 PMC4879420 27021328

[bb23] Singh, M., Bhandary, S., Bhowal, R. & Chopra, D. (2018). *CrystEngComm*, **20**, 2253–2257.

[bb24] Song, R. Q. & Cölfen, H. (2011). *CrystEngComm*, **13**, 1249–1276.

[bb25] Thallapally, P. K., Jetti, R. K., Katz, A. K., Carrell, H. L., Singh, K., Lahiri, K. & Desiraju, G. R. (2004). *Angew. Chem. Int. Ed.* **43**, 1149–1155. 10.1002/anie.200352253 14983460

[bb26] Tong, H. H., Chow, A. S., Chan, H. M., Chow, A. H., Wan, Y. K., Williams, I. D., Shek, F. L. & Chan, C. K. (2010). *J. Pharm. Sci.* **99**, 1942–1954. 10.1002/jps.21983 19894277

[bb27] Wolff, S. K., Grimwood, D. J., McKinnon, J. J., Turner, M. J., Jayatilaka, D. & Spackman, M. A. (2012). *Crystal Explorer.* University of Western Australia.

[bb28] Yeşilada, E. & Küpeli, E. (2002). *J. Ethnopharmacol.* **79**, 237–248. 10.1016/s0378-8741(01)00387-7 11801387

